# The effect of sedation and time after cardiac arrest on coma outcome prognostication based on EEG power spectra

**DOI:** 10.1093/braincomms/fcad190

**Published:** 2023-06-28

**Authors:** Andria Pelentritou, Nathalie Ata Nguepnjo Nguissi, Manuela Iten, Matthias Haenggi, Frederic Zubler, Andrea O Rossetti, Marzia De Lucia

**Affiliations:** Laboratoire de Recherche en Neuroimagerie (LREN), University Hospital (CHUV) & University of Lausanne, 1011 Lausanne, Switzerland; Laboratoire de Recherche en Neuroimagerie (LREN), University Hospital (CHUV) & University of Lausanne, 1011 Lausanne, Switzerland; Department of Intensive Care Medicine, Inselspital, Bern University Hospital, University of Bern, 3010 Bern, Switzerland; Department of Intensive Care Medicine, Inselspital, Bern University Hospital, University of Bern, 3010 Bern, Switzerland; Department of Neurology, Spitalzentrum Biel, University of Bern, 2501 Biel, Switzerland; Department of Clinical Neurosciences, University Hospital (CHUV) & University of Lausanne, 1011 Lausanne, Switzerland; Laboratoire de Recherche en Neuroimagerie (LREN), University Hospital (CHUV) & University of Lausanne, 1011 Lausanne, Switzerland

**Keywords:** coma, cardiac arrest, EEG, early prognostication, sedation

## Abstract

Early prognostication of long-term outcome of comatose patients after cardiac arrest remains challenging. Electroencephalography-based power spectra after cardiac arrest have been shown to help with the identification of patients with favourable outcome during the first day of coma. Here, we aim at comparing the power spectra prognostic value during the first and second day after coma onset following cardiac arrest and to investigate the impact of sedation on prognostication. In this cohort observational study, we included comatose patients (*N* = 91) after cardiac arrest for whom resting-state electroencephalography was collected on the first and second day after cardiac arrest in four Swiss hospitals. We evaluated whether the average power spectra values at 4.6–15.2 Hz were predictive of patients’ outcome based on the best cerebral performance category score at 3 months, with scores ranging from 1 to 5 and dichotomized as favourable (1–2) and unfavourable (3–5). We assessed the effect of sedation and its interaction with the electroencephalography-based power spectra on patient outcome prediction through a generalized linear mixed model. Power spectra values provided 100% positive predictive value (95% confidence intervals: 0.81–1.00) on the first day of coma, with correctly predicted 18 out of 45 favourable outcome patients. On the second day, power spectra values were not predictive of patients’ outcome (positive predictive value: 0.46, 95% confidence intervals: 0.19–0.75). On the first day, we did not find evidence of any significant contribution of sedative infusion rates to the patient outcome prediction (*P* > 0.05). Comatose patients’ outcome prediction based on electroencephalographic power spectra is higher on the first compared with the second day after cardiac arrest. Sedation does not appear to impact patient outcome prediction.

## Teaser

Pelentritou *et al.* demonstrate that electroencephalography-based power spectra can aid in the prognostication of coma outcome on the first day more so than the second day following cardiac arrest. Sedative administration, forming part of the standard clinical care in coma patients, does not appear to influence outcome prediction.

## Graphical Abstract

**Graphical Abstract:**
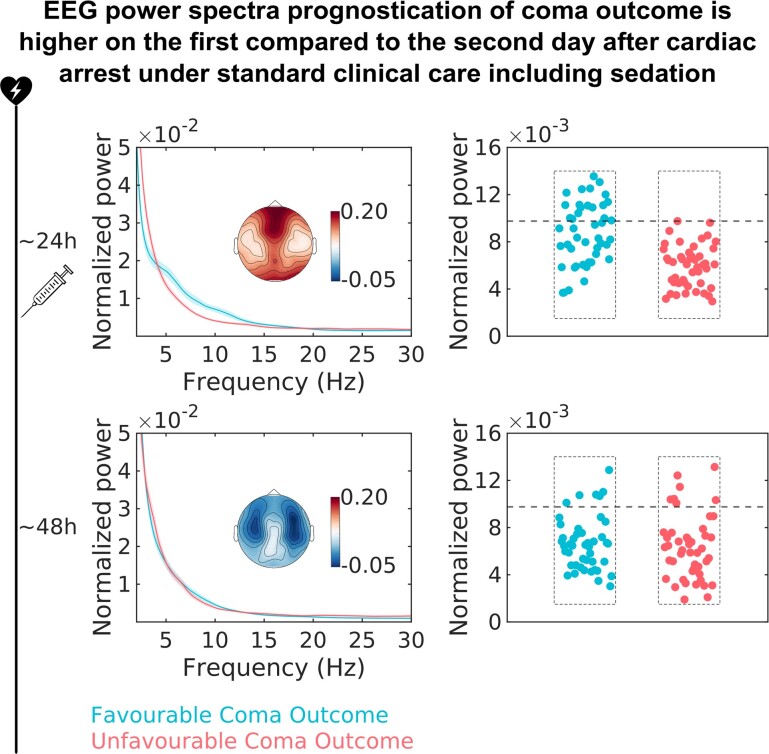

## Introduction

Cardiac arrest (CA) is a major global health problem, causing up to 20% of deaths in western countries.^1^ Advances in resuscitation and post-CA care have allowed for improvement of survival rate and functional outcome.^2^ From those patients admitted to the intensive care unit after CA, many remain unconscious and suffer from post-CA syndromes, known to affect cerebral and cardiac function.^3,4^ Patients are sedated and may be treated with targeted temperature management (TTM) during 12–24 h.^5^ In patients who remain comatose and survive, awakening usually occurs within 5 days after CA.^6^ Outcome prognostication of comatose patients remains a difficult task for clinicians and is currently based on the combined results of a variety of tests, including clinical examination, blood biomarkers, electrophysiological investigations and brain imaging,^7,8^ and often requires multiple examinations over the first days after coma onset. Despite a combination of EEG markers and clinical examinations increasing the accuracy of prognostication of unfavourable outcome (UO), many patients remain in a ‘grey zone’, where outcome prediction can be difficult.^9,10^ Improvements in long-term outcome prognostication on the first day after coma onset would aid in informing clinicians and relatives, encourage the development of early medical intervention and optimize intensive treatment allocation.

Prognostic markers based on EEG recordings are commonly used as part of the clinical routine and include the evaluation of the background activity, reactivity and the presence of epileptiform features.^7,9,11^ In assessing the reliability of these EEG markers, some studies suggested that the highest positive predictive value (PPV) can be observed within 24 h after CA,^12,13^ after which EEG features evolve to less specific patterns.^9^ Classically, the majority of standardized clinical tests are evaluated in relation to the prediction of poor outcome;^7,8^ however, more recently, attention towards good outcome prognostication has emerged.^14,15^ A previous electrophysiological study performed with this goal, showed that the power spectral analysis of resting EEG acquired within the first day after CA can provide a quantitative marker for outcome prediction and is complementary to EEG reactivity.^16^ This study showed maximal PPV for favourable outcome (FO) patients by averaging the EEG power at 5.2–13.2 Hz, possibly reflecting a higher degree of preserved cortico-thalamic connections in patients with good prognosis.^16,17^ Complementing these previous investigations, here we aimed at shedding light on how the power spectral features may be influenced by the passage of time after coma onset, and by sedation, typically administered during the first days after coma onset and known to affect the EEG power in the frequency range of interest.^18^ Although a controlled study on the effect of time and sedation would require the inclusion of patients with and without sedation, the current clinical practice precludes such a controlled experimental design. Instead, considering the current clinical guidelines, we included patients whose EEG could be recorded during the first and second day after CA, all of which received similar clinical intervention, and we attempted at disentangle the effect of sedation and passage of time through a set of multivariate statistical analyses.

## Materials and methods

### Patient population

EEG recordings were acquired on 138 comatose patients (minimum age: 18 years), resuscitated after CA and admitted to the intensive care units of the University Hospitals of Lausanne (*N* = 68), Sion (*N* = 5), Fribourg (*N* = 2) and Bern (*N* = 63) in Switzerland, between July 2014 and January 2018.^16^ TTM was part of the clinical management in all recruitment centres. Patients from all centres (*N* = 138) were recorded within the first 24 h after CA, they were sedated and most of them were treated with TTM (*N* = 133) at 33°C (*N* = 18), or 36°C (*N* = 115), or no intervention (*N* = 5). TTM was followed by slow rewarming and halting of TTM during the next 24 h. Sedation was administered via continuous infusion and bolus injections to allow adequate mechanical ventilation during at least the first 24 h after CA.^7,19,20^ The analyses of EEG data acquired on the first day of coma have been previously reported.^16^ For a subset of patients (*N* = 91), a second EEG recording was acquired on the second day after coma onset. Acquisition of a second recording was not performed when patients awoke or died rapidly after the first recording or when access to the patient was prevented by urgent medical intervention or for logistic reasons, such as unavailability of the experimenter and/or of the necessary equipment.

### Clinical assessment

The Full Outline of UnResponsiveness score was used to assess the patients’ functional and neurological condition at both time points of EEG data acquisition.^21^ A certified neurologist assessed brainstem reflexes and motor reactivity to pain stimulation after weaning of sedation at around 72 h.^22^ If clinically indicated, bilateral median nerve somatosensory evoked potentials were performed between 24 and 48 h after CA. Withdrawal of intensive care was decided using a multimodal approach at 72 h after CA and considered when at least two of the following criteria were evident: incomplete return of the pupillary and corneal reflexes, treatment-resistant myoclonus, bilateral absence of cortical somatosensory evoked potentials when available and unreactive EEG background activity after sedation weaning.^22,23^ Brain MRI, serum-neuron-specific enolase represented additional prognostic modalities in unclear cases. Clinical decisions were independent of the results of the EEG power spectral analysis,^16^ as clinicians were blinded to them.

Patient outcome was assessed using the cerebral performance category and dichotomized to FO (cerebral performance category of 1–2) and UO (cerebral performance category of 3–5).^24^ This was based on the best score within 3 months after CA (routine phone call with a semi-structured interview), which took into consideration the cerebral performance category values, outcome at hospital discharge and neurological examination during hospitalization.

### EEG data acquisition and preprocessing

Resting-state EEG recordings were acquired at bedside, in the intensive care unit. First and second day recordings were carried out within 28 h after estimated CA (referred to as first day) and between 28 and 56 h after estimated CA (referred to as second day), respectively. During each recording, resting EEG was recorded for 8–20 min using a 63 active ring electrode array (g.Hiamp, g.tec medical engineering, Graz, Austria), arranged according to the international 10–10 system with a sampling rate of 1200 Hz and referenced to the right ear lobe. Vertical and horizontal electrooculography were acquired using single-use Ag/AgCl attached below the eye and to the outer right canthus, respectively. Electrocardiography was acquired using additional electrodes attached to the patient’s chest.

The same EEG data preprocessing was performed on the recordings acquired on the first and second day as previously described.^16^ Data analyses were performed in MATLAB (R2019b, The MathWorks, Natick, MA, USA) using an open-source toolbox, FieldTrip (version 20201205^25^) and custom-made scripts. The raw EEG data were band-pass filtered between 1 and 40 Hz, segmented offline into 5 s epochs and down-sampled to 500 Hz.^16^ Components containing an identifiable electro-oculographic and electro-cardiographic signal were identified using Independent Component Analysis^25,26^ and removed and any artefactual epochs were excluded from further analysis. Artefactual electrodes were interpolated from neighbouring electrodes, and average reference was applied.

### EEG power spectral analysis

Power spectra analyses were performed on patients for which a recording on Day 1 and a recording on Day 2 were available (*N* = 91). Power spectra were computed for each patient in each 5 s epoch and electrode between 2 and 40 Hz in 0.2 Hz steps, based on nine Slepian tapers for 1 Hz smoothing, as implemented in FieldTrip.^25^ For each recording day, power spectra were then averaged across epochs, and results were normalized based on the sum of all spectral values.^16^ This step resulted in an average power spectrum across frequencies at each electrode for each patient and recording.

### EEG power spectra statistical analysis and outcome prediction

For patient outcome prediction, power spectra values and specific parameters were optimized using a 5-fold cross-validation procedure as explained below. The same analysis was carried out separately for the first and second day power spectra.

In each of the five folds, 80% of the patients were used as the training dataset, and 20% as the test dataset. In each training and test dataset, we included matched numbers of FO and UO patients. For each training dataset, power spectra values of included patients were utilized for identifying the frequency range at which the power spectra of FO patients differed from those of UO patients based on a cluster permutation statistical test. Cluster-based permutation consists of a non-parametric test allowing for multiple comparisons correction in space (i.e. across EEG electrodes) and frequency; in this study using 5000 permutations and a two-sided cut off at *P* < 0.05.^16,25,27^ In each fold, patient outcome prediction was based on the average of the spectral values across all electrodes and within the range of frequencies which were discriminative (*P* < 0.05, two-tailed) between FO and UO and on a threshold value that achieved maximal PPV. The test dataset was used for an unbiased evaluation of the patient outcome prediction based on the frequency band and threshold values identified during training. The test datasets were non-overlapping, and each patient’s power spectra value was tested only once.

Finally, for the first and second day, we evaluated the coma outcome prediction based on the combination of the power spectra results and the available clinical scores of EEG discontinuity and EEG reactivity. Of note, the prediction results based on the combination of the EEG power spectra values and these clinical scores were not independent of the clinical decision on the patient management, since the latter formed part of the metrics utilized in making such decision.

### Demographics statistical analysis

In addition to the EEG recordings, we collected patient demographics as well as the values of time to Return of Spontaneous Circulation, aetiology and type of CA. We measured the time from CA to first day EEG and second day EEG, body core temperature, sedation infusion rates (propofol, midazolam, and fentanyl), and we collected the clinical EEG reactivity, EEG discontinuity, the presence of repetitive epileptiform activity, the score of the best pupillary and corneal reflexes and motor response evaluated within 48 h. The motor response was dichotomized based on the Glasgow motor response score as present (motor response: 1, 2) or absent (motor response: 3 or higher).

For descriptive purposes, a statistical assessment of demographic information was performed between FO and UO patients and between FO patients with correctly predicted versus incorrectly predicted outcome. Statistical analysis was performed using non-parametric Kruskal–Wallis one-way ANOVAs or non-parametric two-sided Fisher’s exact tests for the comparison of subject numbers (statistical significance set at *P* = 0.05). Statistical analyses were performed only on the subset of patients recorded on both days (*N* = 91).

### Sedation statistical analysis

The following comparisons were performed to investigate whether differences in the sedation regimen could explain the power spectra-based outcome prediction results during each day. We compared the sedation infusion rates using non-parametric Kruskal–Wallis one-way ANOVAs and the number of patients under sedation using Fisher’s exact tests (statistical significance set at *P* = 0.05). These comparisons were conducted between the first and second day, between FO and UO patients and finally between FO patients with correctly predicted versus incorrectly predicted outcome.

In order to further investigate the impact of sedation in combination with the EEG power spectra, we computed a logistic binomial model of the probability of observing FO or UO based on the EEG power spectra value and the infusion rates for each of three sedative agents. This generalized linear mixed model was fit to the data using the *fitglm* function, as implemented in MATLAB (https://ch.mathworks.com/help/stats/fitglm.html). We generated the following four models. In the first model, we considered as independent variable the EEG power spectra, the infusion rate for each sedative agent (Propofol, Midazolam, and Fentanyl) and the interaction between the EEG power spectra and each of the sedative infusion rates. For the remaining three models, we simplified the first by excluding the interaction between the EEG power spectra and one sedative infusion rate (i.e. in the first simplified model, we excluded the interaction between EEG power spectra and Fentanyl, in the second, we excluded the interaction between EEG power spectra and Midazolam and in the third, we did not consider the interaction between EEG power spectra and propofol). Model selection was based on the Akaike Information Criterion (AIC).^28^

### Ethics

The study was approved by the Ethics Committee of the respective institutions [PB_2016-00530 (23/05)]. An informed consent was signed prior to data acquisition by a family member, a legal representative or a treating clinician not involved in the study.

## Results

### Patient characteristics

From 138 patients recorded during the first day of coma, 80 had FO. From the subset of 91 patients for whom recordings were acquired on both the first and second day, 45 had FO and 46 had UO. These 91 patients received TTM at either 33°C (*N* = 8), at 36°C (*N* = 80), or no intervention (*N* = 3) during the first day of coma.

### Power spectra and outcome prediction

**Figure 1:**
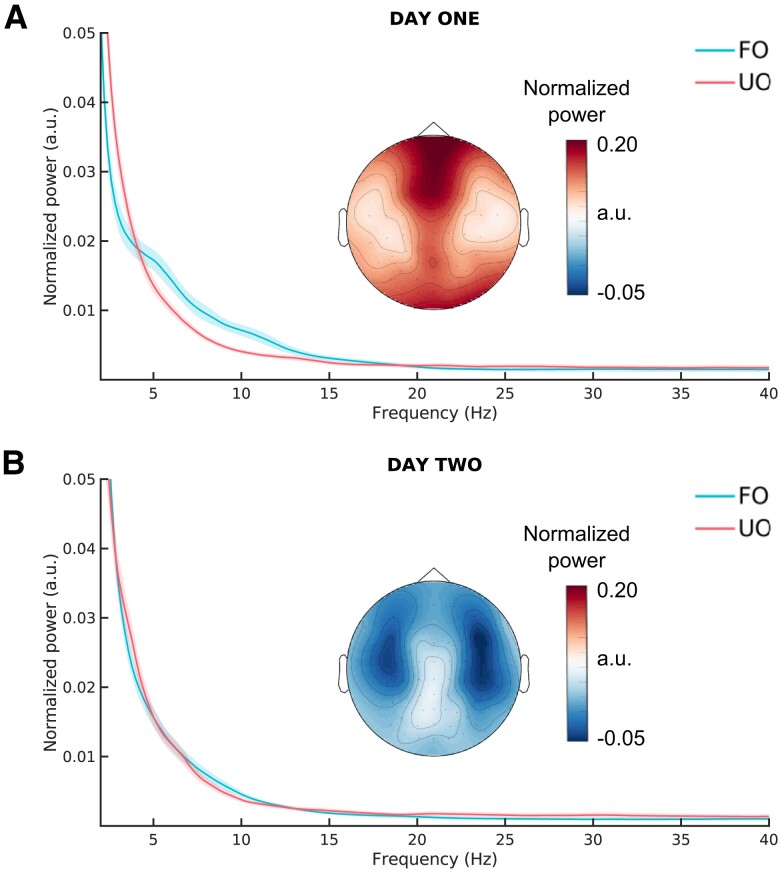
**Power spectra for patients on Days 1 and 2 after coma onset.** Normalized power spectra frequencies, averaged across patients and across electrodes, in arbitrary units for patients in the training set of one representative fold with FO, blue line and UO, pink line for Days 1 and 2 (*N* = 73). On Day 1, differences were observed across the whole electrode montage and especially between 4.6 and 15.2 Hz (cluster permutation statistical analysis, *P* < 0.05, two-tailed). Topographical insets illustrate the difference in power spectra between FO and UO in the frequency range between 4.6 and 15.2 Hz

In the first day of coma, the cluster permutation statistical analysis was performed for each of the five training sets and produced significant differences (*P* < 0.05, two-tailed) along a similar range of frequencies (e.g. 4.6–15.2 and 4.6–15.4 Hz, Supplementary Table 1). In each training set, averaged power spectra values for each patient were generated based on the range of frequencies that were significantly different in each fold and a power spectra threshold was defined based on the maximum power spectrum value that correctly classified all UO patients. The resulting five thresholds were then applied to the corresponding test sets. This yielded similar results across the five test sets with an average classification (mean ± standard deviation) accuracy of 0.70 ± 0.01 across folds and average PPV of 0.99 ± 0.02, negative predictive value (NPV) of 0.63 ± 0.00, sensitivity of 0.40 ± 0.01 and specificity of 1.00 ± 0.01 (Supplementary Table 1). For the first day, since all folds produced largely similar results over a similar range of discriminative frequencies, we chose one representative fold with significant differences in the range of frequencies between 4.6 and 15.2 Hz across all electrodes (*P* < 0.05, two-tailed; Fig. 1A).

The same analysis on the second day after coma onset, failed to produce significant differences between the training sets of FO and UO in all cross-validation folds (Supplementary Table 1). Therefore, to allow for a comparison of the prediction performance between the first and second day, for the second day, we computed the average spectral values between 4.6 and 15.2 Hz (Fig. 1B), as was applied on the first day. Similarly, the threshold for the discrimination of FO and UO selected based on one representative fold on the first day was applied to the second day.

**Figure 2:**
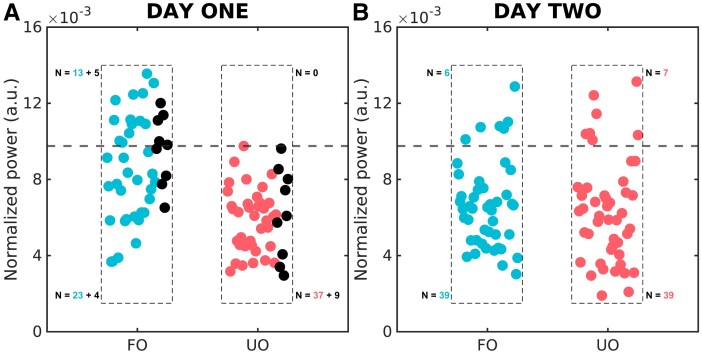
**Prognostication of coma outcome based on power spectra on Days 1 and 2 after coma onset.** Outcome prediction based on normalized spectral power in arbitrary units for Days 1 and 2. Blue dots show patients in the training set for one representative fold with FO; *N* = 36, pink dots show patients in the training with UO; *N* = 37 and black dots show patients belonging in the test set for one representative fold (FO: *N* = 9; UO: *N* = 9). The dashed line indicates the threshold for outcome prediction; values above the threshold should predict FO. For Day 1, in the subset of patients recorded on both days, we correctly predicted FO in 18 patients without false positives. For Day 2, applying the same threshold as Day 1, we found no significant outcome prediction

On Day 1, among the 91 patients, 18 out of 45 FO patients were correctly predicted [PPV: 1.00, 95% confidence interval (CI): 0.81–1.00; Fig. 2A; Table 1]. On Day 2, outcome prediction was at chance level (PPV: 0.46, 95% CI: 0.19–0.75; Fig. 2B; Table 2). For the first day, investigating the predictive value of EEG reactivity, EEG discontinuity and the additive prediction of these values in combination with the power spectra, we observed that the combination of the EEG power spectra and discontinuity provided higher predictive value (PPV: 0.83, 95% CI: 0.67–0.93) compared with each of them separately (Table 1). In addition, we observed that EEG reactivity (PPV: 0.88, 95% CI: 0.74–0.96) and the combination of power spectra and EEG reactivity (PPV: 0.88, 95% CI: 0.76–0.94) provided the most accurate prediction (Table 1). Similar to the first day, on the second day (Table 2), the EEG reactivity alone provided the highest PPV (0.76, 95% CI: 0.61–0.87) and accuracy (0.81, 95% CI: 0.70–0.89) however, unlike the first day, the EEG reactivity prognostication performance was reduced by its combination to the power spectra values (PPV: 0.72, 95% CI: 0.58–0.84; accuracy: 0.75, 95% CI: 0.65–0.83).

**Table 1:** Prediction results for FO on Day 1, based on power spectra, EEG discontinuity and EEG reactivity, separately and in combination^a^

Day 1						
Predictor	TP/FP/FN/TN	PPV	NPV	Sensitivity	Specificity	Accuracy
Power spectra (4.6–15.2 Hz)	18/0/27/46	1.00 (0.81–1.00)	0.63 (0.51–0.74)	0.40 (0.26–0.56)	1.00 (0.92–1.00)	0.70 (0.60–0.79)
EEG discontinuity	30/7/14/33	0.81 (0.65–0.92)	0.70 (0.55–0.83)	0.68 (0.52–0.81)	0.83 (0.67–0.93)	0.75 (0.64–0.84)
Combination of power and EEG discontinuity	33/7/12/39	0.83 (0.67–0.93)	0.76 (0.63–0.87)	0.73 (0.58–0.85)	0.85 (0.71–0.94)	0.79 (0.69–0.87)
EEG reactivity	31/5/8/31	0.88 (0.74–0.96)	0.79 (0.64–0.91)	0.79 (0.64–0.91)	0.86 (0.71–0.95)	0.83 (0.72–0.90)
Combination of power and reactivity	37/5/8/41	0.88 (0.76–0.94)	0.84 (0.70–0.93)	0.82 (0.68–0.92)	0.89 (0.76–0.96)	0.86 (0.77–0.92)

a 95% CIs are shown in parentheses.

TP, true positive; FP, false positive; FN, false negative; TN, true negative; PPV, positive predictive value; NPV, negative predictive value.

**Table 2:** Prediction results for FO on Day 2, based on power spectra, EEG discontinuity and EEG reactivity, separately and in combination^a^

Day 2						
Predictor	TP/FP/FN/TN	PPV	NPV	Sensitivity	Specificity	Accuracy
Power spectra (4.6–15.2 Hz)	6/7/39/39	0.46 (0.19–0.75)	0.50 (0.38–0.62)	0.13 (0.05–0.27)	0.85 (0.71–0.94)	0.49 (0.39–0.60)
EEG discontinuity	38/20/2/16	0.66 (0.52–0.78)	0.89 (0.65–0.99)	0.95 (0.83–0.99)	0.44 (0.28–0.62)	0.71 (0.60–0.81)
Combination of power and EEG discontinuity	39/22/6/24	0.64 (0.51–0.76)	0.80 (0.61–0.92)	0.87 (0.73–0.95)	0.52 (0.37–0.67)	0.69 (0.59–0.78)
EEG reactivity	35/11/3/25	0.76 (0.61–0.87)	0.89 (0.72–0.98)	0.92 (0.79–0.98)	0.69 (0.52–0.84)	0.81 (0.70–0.89)
Combination of power and reactivity	36/14/9/32	0.72 (0.58–0.84)	0.78 (0.62–0.89)	0.80 (0.65–0.90)	0.70 (0.54–0.82)	0.75 (0.65–0.83)

a 95% CIs are shown in parentheses.

TP, true positive; FP, false positive; FN, false negative; TN, true negative; PPV, positive predictive value; NPV, negative predictive value.

### Clinical characteristics and outcome prediction

First, we compared clinical characteristics of patients with FO and UO (Supplementary Table 2), and we identified statistically significant differences in numerous of those including pupillary reflexes (*P* < 0.005), corneal reflexes (*P* < 0.005) and motor responses (*P* < 0.0005). Evaluating each day separately, we found statistically significant differences in the clinical scores of EEG discontinuity (*P* < 0.0005), EEG reactivity (*P* < 0.0005) and EEG epileptiform activity (*P* < 0.0005). Second, for the first day, we compared the clinical characteristics of FO patients that were correctly versus incorrectly predicted (Supplementary Table 3), identifying no significant differences in all patient characteristics (*P* > 0.05), except for EEG discontinuity (*P* < 0.05). Finally, for the second day, we contrasted the clinical characteristics of FO and UO patients that were correctly versus incorrectly predicted (using the power spectra threshold from the first day outcome prognostication), which revealed no statistically significant differences for any of the comparisons (*P* > 0.05).

### Sedation and outcome prediction

In order to investigate the impact on sedation on outcome classification, first, we compared sedative infusion rates and the number of patients under sedation between Days 1 and 2 (Table 3). In patients with FO, we identified a significantly higher number of patients sedated with midazolam (*P* < 0.0005) and fentanyl (*P* < 0.0005) on the first versus the second day. In patients with UO, we observed a significantly higher number of patients sedated with propofol (*P* < 0.05), midazolam (*P* < 0.005) and fentanyl (*P* < 0.0005) on the first compared with the second day. Finally, there was a statistically significant higher number of patients sedated with propofol (*P* < 0.05), midazolam (*P* < 0.0005) and fentanyl (*P* < 0.0005) on the first day compared the second day when considering all patients irrespective of their outcome.

**Table 3:** Comparison of sedation concentrations, number of sedated patients, and concomitant sedation between Days 1 and 2 in the subset of patients recorded on both days (*N* = 91)^a^

	Day 1	Day 2	*P*-value	Test
FO *N*	45	45		
Propofol (mg/kg/h)	2.46 ± 1.23	2.17 ± 1.40	0.36	Kruskal–Wallis
	34 (2)	27 (2)	0.15	Fisher
Midazolam (mg/kg/h)	0.13 ± 0.10	0.09 ± 0.05	0.46	Kruskal–Wallis
	20 (2)	4 (2)	<0.0005	Fisher
Fentanyl (μg/kg/h)	0.60 ± 0.67	1.31 ± 1.60	<0.05	Kruskal–Wallis
	37 (2)	19 (2)	<0.0005	Fisher
Number of sedatives				
Zero	0 (2)	11 (2)	<0.0005	Fisher
One	4 (2)	17 (2)	<0.005	Fisher
Two	30 (2)	12 (2)	<0.0005	Fisher
Three	9 (2)	3 (2)	0.12	Fisher
UO *N*	46	46		
Propofol (mg/kg/h)	2.32 ± 1.24	2.19 ± 1.46	0.65	Kruskal–Wallis
	28 (0)	16 (0)	<0.05	Fisher
Midazolam (mg/kg/h)	0.12 ± 0.12	0.20 ± 0.24	0.37	Kruskal–Wallis
	18 (0)	5 (0)	<0.005	Fisher
Fentanyl (μg/kg/h)	0.81 ± 0.64	0.95 ± 0.75	0.82	Kruskal–Wallis
	28 (0)	9 (0)	<0.0005	Fisher
Number of sedatives				
Zero	3 (0)	25 (0)	<0.0005	Fisher
One	15 (0)	13 (0)	0.82	Fisher
Two	25 (0)	7 (0)	<0.0005	Fisher
Three	3 (0)	1 (0)	0.62	Fisher
ALL *N*	91	91		
Propofol (mg/kg/h)	2.40 ± 1.23	2.18 ± 1.41	0.34	Kruskal–Wallis
	62 (2)	43 (2)	<0.05	Fisher
Midazolam (mg/kg/h)	0.12 ± 0.11	0.15 ± 0.18	0.85	Kruskal–Wallis
	38 (2)	9 (2)	<0.0005	Fisher
Fentanyl (μg/kg/h)	0.88 ± 0.63	1.35 ± 1.42	0.22	Kruskal–Wallis
	65 (2)	28 (2)	<0.0005	Fisher
Number of sedatives				
Zero	3 (2)	36 (2)	<0.0005	Fisher
One	19 (2)	30 (2)	0.09	Fisher
Two	55 (2)	19(2)	<0.0005	Fisher
Three	12 (2)	4 (2)	0.06	Fisher

a Sedation infusion rates are depicted as mean ± standard deviation next to the sedative agent names. The number of patients for which the statistic is computed is shown with the number of patients for which the value is missing in parentheses.

Subsequently, we compared the sedative infusion rates and the number of patients under sedation between FO and UO during the first and second day (Table 4). We found a statistically significant higher number of FO patients sedated with propofol (*P* < 0.05) on the second day and sedated with fentanyl (*P* < 0.05) on both the first and second days.

**Table 4:** Comparison of sedation concentrations, number of sedated patients and concomitant sedation between FO and UO in the subset of patients recorded on both days (*N* = 91)^a^

	FO	UO	*P*-value	Test
Day 1 *N*	45	46		
Propofol (mg/kg/h)	2.46 ± 1.23	2.32 ± 1.24	0.57	Kruskal–Wallis
	34 (2)	28 (0)	0.07	Fisher
Midazolam (mg/kg/h)	0.13 ± 0.10	0.12 ± 0.12	0.35	Kruskal–Wallis
	20 (2)	18 (0)	0.53	Fisher
Fentanyl (μg/kg/h)	0.60 ± 0.67	0.81 ± 0.64	0.18	Kruskal–Wallis
	37 (2)	28 (0)	<0.05	Fisher
Number of sedatives				
Zero	0 (2)	3 (0)	0.24	Fisher
One	4 (2)	15 (0)	<0.005	Fisher
Two	30 (2)	25 (0)	0.19	Fisher
Three	9 (2)	3 (0)	0.06	Fisher
Day 2 *N*	45	46		
Propofol (mg/kg/h)	2.17 ± 1.40	2.19 ± 1.46	0.91	Kruskal–Wallis
	27 (2)	16 (0)	<0.05	Fisher
Midazolam (mg/kg/h)	0.09 ± 0.05	0.20 ± 0.24	0.62	Kruskal–Wallis
	4 (2)	5 (0)	1.00	Fisher
Fentanyl (μg/kg/h)	1.31 ± 1.60	0.95 ± 0.75	0.48	Kruskal–Wallis
	19 (2)	9 (0)	<0.05	Fisher
Number of sedatives				
Zero	11 (2)	25 (0)	<0.05	Fisher
One	17 (2)	13 (0)	0.27	Fisher
Two	12 (2)	7 (0)	0.20	Fisher
Three	3 (2)	1 (0)	0.35	Fisher

a Sedation infusion rates are depicted as mean ± standard deviation next to the sedative agent names. The number of patients for which the statistic is computed is shown with the number of patients for which the value is missing in parentheses.

Finally, to investigate the impact of sedation on outcome prediction, we contrasted the number of patients under sedation and the sedation infusion rates in FO patients that were correctly versus incorrectly predicted on the first day, which revealed no statistically significant differences (Table 5).

**Table 5:** Comparison of sedation concentrations and number of sedated patients between FO correctly versus incorrectly predicted on Day 1, in the subset of FO patients recorded on both days (*N* = 45)^a^

	Power spectra			
	Correct prediction	Incorrect prediction	*P*-value	Test
FO *N*	18	27		
Propofol (mg/kg/h)	2.58 ± 1.69	2.38 ± 0.83	0.97	Kruskal–Wallis
	14 (1)	20 (1)	1.00	Fisher
Midazolam (mg/kg/h)	0.12 ± 0.06	0.13 ± 0.12	0.62	Kruskal–Wallis
	8 (1)	12 (1)	1.00	Fisher
Fentanyl (μg/kg/h)	0.85 ± 0.43	1.01 ± 0.75	0.87	Kruskal–Wallis
	17 (1)	20 (1)	0.07	Fisher

a Sedation infusion rates are depicted as mean ± standard deviation next to the sedative agent names. The number of patients for which the statistic is computed is shown with the number of patients for which the value is missing in parentheses.

In order to further characterize the impact of sedation in relation to the EEG power spectra, we computed binomial logistic regression models attempting to address the interaction between each of the sedative agents and the power spectra on the patient outcome. We computed four models investigating this interaction (see Sedation statistical analysis section). The corresponding AIC values indicated that two out of the four models performed equally well with lower AIC: the model with interaction terms EEG power spectra X Propofol infusion rates and EEG power spectra X Midazolam infusion rates (AIC = 97.98) and the model with interaction terms EEG power spectra X Propofol infusion rates and EEG power spectra X Fentanyl infusion rates (AIC = 97.57). We therefore excluded the model with interaction terms: EEG power spectra X Propofol infusion rates, EEG power spectra X Midazolam infusion rates and EEG power spectra X Fentanyl infusion rates (AIC = 99.35) and the model with interaction terms EEG power spectra X Midazolam infusion rates and EEG power spectra X Fentanyl infusion rates (AIC = 99.55).

The first model considering the interaction term with propofol and midazolam produced significant discrimination of patient outcome only based on EEG power spectra (*t* = 2.28, *P* = 0.02), all other predictors and interaction terms yielded no significant results (*P* > 0.05). In the second model including the interactions with propofol and fentanyl, no variables yielded significant results (*P* > 0.05).

## Discussion

We investigated the predictive performance of EEG power spectra in predicting comatose patients’ outcome after CA during the first 2 days of coma. As expected from previous results,^16^ the average power spectra between 4.6 and 15.2 Hz were highly predictive of FO on the first day of coma (PPV: 1.00, 95% CI: 0.81–1.00; Fig. 2A; Table 1). Of note, from a clinical point of view, the present study validates the high PPV of the EEG power spectra on the first day, since the outcome prediction is confirmed even upon exclusion of the extreme cases of patients who passed away or woke up soon after the first recording. In addition, we showed that in the same cohort of patients, the EEG power spectra on the second day after coma onset were not informative of FO (Figs 1 and 2). Only on the first day of coma, EEG power spectra prediction was complementary to that obtained based on EEG reactivity or discontinuity, providing higher accuracy when considering the combination of EEG power spectra with these markers in comparison to each separately (Table 1).

### Power spectra allow coma outcome prognostication only on the first day

Our results on the power spectra-based outcome prediction are consistent with previous studies showing that EEG pattern analyses offer a higher predictive value when performed on data acquired within the first 24 h post-CA compared with later stages.^9,12,13,22^ These results complement previous evidence of the predictive value of early EEG recordings as shown by the analysis of functional connectivity,^29^ of the neural responses to auditory stimulation,^30^ and by the progression of auditory responses during the first 2 days after coma onset.^15,31^ The highly predictive value for FO using the average power spectra at 4.6–15.2 Hz is also reminiscent of a recent study investigating a population with acute consciousness impairment of various aetiologies where, independent of the time of recording from coma onset, spindles, at 12–16 Hz, were predictive of FO.^32^ The high predictive value for the first day of coma can be related to the chronological evolution of the complex pathophysiology of the post-anoxic-ischaemic brain, which is characterized by four phases: an acute phase, within 24 h after hypoxia; an early subacute phase, from Days 1 to 13; a late subacute phase, from Days 14 to 21; and a chronic phase from Day 21, associated with physiological changes.^33^ In particular, from the acute to the early subacute phase, some studies described an evolving pathophysiology with possible occurrence of cortical laminar necrosis.^32^

The higher EEG power on the first compared with the second day of coma appears in contradiction with previous reports in disorder of consciousness patients wherein pronounced alpha peaks were only observed in patients emerging from a minimally conscious state and associated with the highest level of anterior forebrain cortico-thalamic integrity (the ‘D’ type, as is referred to by the authors).^17,34^ In addition, none of the comatose patients with FO reported in this previous study were categorized as ‘D-type’,^17^ in contrast to the pronounced alpha peaks identified in patients with FO in our study (see also Supplementary Fig. 1 in our previous publication^16^). Although these inconsistent results might be due to differences in sedation, TTM and/or the latency at which patients were recorded after coma onset, they also demonstrate that the presence of a prominent EEG power peak in the alpha range is not always related to consciousness level, as has been previously suggested for disorders of consciousness patients,^35^ at least in this acute phase.

### Sedation regimen does not influence coma outcome prognostication

As expected, we found that a higher number of patients were sedated on Day 1 compared with Day 2 (Table 3), consistent with common practices of waning the sedation administration in the intensive care unit after the first day.^7^ In terms of the comparison between FO and UO patients, we only found differences in the number of patients sedated with fentanyl (on both days) and propofol (on the second day). Effects of these agents in the range of frequencies of interest (4.6–15.2 Hz; Fig. 1) have been amply documented in healthy volunteers. Remifentanyl, a commonly employed sedative analgesic in the clinics, has previously been shown to alter the EEG in the theta and alpha range (0.5–12 Hz)^36^ and graph theoretical measures in the alpha and low beta range (8–18 Hz).^37^ In addition, propofol can induce modulation of the EEG power^38,39^ and functional connectivity^40^ in the alpha range. Given these known effects of sedative agents, the EEG power spectra difference between FO and UO patients on the first day could be driven by the effect of fentanyl rather than the severity of their clinical condition, leading to their outcome.

The absence of significant differences in the number of FO patients under sedation (Table 5) that were correctly versus incorrectly predicted on the first day suggests that prognostication on the first day is not a direct consequence of the sedation regimen, at least within the frequency range of interest (4.6–15.2 Hz), in line with previous reports.^13^ In addition, the absence of significant differences in the EEG power spectra on the second day despite higher number of FO compared with UO-sedated patients, further suggests that sedation did not induce a measurable effect on the EEG in our cohort. In agreement with the above, we did not find evidence of any significant contribution of the sedative infusion rates nor of the interaction between the EEG power spectra and the sedative infusion rates in predicting patient outcome, as uncovered by a multivariate logistic regression model. On the other hand, given that the administration of several concomitant sedative agents (Tables 3 and 4) can produce complex interaction effects,^13,41,42^ the question of whether the concomitant administration of sedative agents may have a major role on the prognostic value of the EEG power spectra on the first day remains open.

### Limitations

The choice of sedation, TTM and latency at which we recorded the EEG was a consequence of the clinical management regimes and logistic reasons, which were independent from our study goals and prevented the investigation of this patient population as part of a controlled study. Therefore, we were not able to assess the effect of each of these factors separately and in a systematic manner, for example by assessing the effect of sedative infusion rates on the EEG power spectra as a continuum over time. This limitation could be partially addressed by including a larger cohort of patients and by considering a fine-grained evaluation of the effect of the latency of recording (e.g. at 9 h, 12 h, etc. after CA), of the body temperature, and of the sedative type and infusion rate, which we will consider in future studies.

## Conclusion

We showed that the EEG power spectra provide a more valuable outcome prediction information within the first day post-CA than later and especially so in predicting FO. These results complement previous observations on the predictive value of EEG power spectra for outcome prognostication during the acute phase following traumatic brain injury,^43^ and in chronic DoC patients.^44–47^ Our results suggest that sedation does not act as a major confounder for the observed power spectra difference between FO and UO patients on the first coma day; however, the reason for the decrease in prediction accuracy between the first and second day remains unclear. One possible explanation is that the EEG power spectra during the first compared with the following days reflect different stages in the clinical evolution of the patients’ state, which prevents a straightforward comparison between the neural activity of the first and second day. Understanding the relationship between these different stages in pathophysiological evolution and evidence derived from non-invasive investigations remains an open key point for better identifying the optimal latency after coma onset for coma outcome prediction through EEG quantitative analysis.^48^

## Supplementary Material

fcad190_Supplementary_Data

## Data Availability

The data that support the findings of this study are available from the corresponding author upon reasonable request. Custom-made scripts used in the data analysis are publicly available at https://github.com/DNC-EEG-platform/powerspectracoma.
