# Supplementary material for: The effect of sedation and time after cardiac arrest on coma outcome prognostication based on EEG power spectra
Source: Brain Commun. 2023 Jun 28;5(4):fcad190. doi: 10.1093/braincomms/fcad190 (PMC10353761; doi:10.1093/braincomms/fcad190)
Supplement: fcad190_Supplementary_Data [file fcad190_supplementary_data.docx]

Supplementary Material

**The effect of sedation and time after cardiac arrest on coma outcome prognostication based on EEG power spectra**

Andria Pelentritou*, Nathalie Ata Nguepnjo Nguissi*, Manuela Iten, Matthias Haenggi, Frederic Zubler, Andrea O. Rossetti, Marzia De Lucia

**Supplementary Table 1. Power spectra frequency ranges of interest and predictive performance in all cross-validation folds.**

| **DAY ONE** | | | | | |
| --- | --- | --- | --- | --- | --- |
|  | **FOLD 1** | **FOLD 2** | **FOLD 3** | **FOLD 4** | **FOLD 5** |
| Frequency (Hz) | 4.6 – 15.2 | 4.6 – 15.4 | 4.6 – 15.0  19.0 – 40.0 | 4.6 – 15.6 | 4.4 – 14.0 |
| Test. TP/FP/FN/TN | 5/0/4/9 | 3/1/6/9 | 1/0/8/9 | 4/0/5/9 | 5/0/4/9 |
| **DAY TWO** | | | | | |
|  | **FOLD 1** | **FOLD 2** | **FOLD 3** | **FOLD 4** | **FOLD 5** |
| Frequency (Hz) | 20 - 40 | n.s. | n.s. | n.s | 13.2 – 40 |
| Test. TP/FP/FN/TN | 0/1/45/45 | N/A | N/A | N/A | 0/0/45/46 |

Frequency entries indicate the range of frequencies at which statistically significant differences were identified between FO and UO patients using cluster permutation statistical analysis (p<0.05, two-tailed). TP = True positive; FP = False Positive; FN = False Negative; TN = True Negative; PPV = Positive Predictive Value; NPV = Negative Predictive Value; n.s.= not significant; N/A: not applicable.

**Supplementary Table 2. Demographics and clinical characteristics of FO and UO patients in the subset of patients recorded on both days (N = 91).**

|  | **FO** | **UO** | **p-value** |
| --- | --- | --- | --- |
| *N* | 45 | 46 |  |
| Time to ROSC (min), *M* ± *SD (N_missing_)* | 18.49 ± 12.73 (0) | 28.89 ± 13.30 (0) | **<0.0005** |
| Age (y), *M* ± *SD (N_missing_)* | 63.51 ± 13.44 (0) | 66.20 ± 12.64 (0) | 0.27 |
| Gender (male), *N (N_missing_)* | 39 (0) | 34 (0) | 0.19 |
| Cardiac etiology of CA, *N (N_missing_)* | 34 (0) | 34 (0) | 1.00 |
| Pupillary reflexes, *N (N_missing_)* | 38 (5) | 27 (6) | **<0.005** |
| Corneal reflexes, *N (N_missing_)* | 32 (5) | 18 (6) | **<0.005** |
| Motor response, *N (N_missing_)* | 27 (5) | 5 (8) | **<0.0005** |
| Time to EEG (h): day one, *M* ± *SD (N_missing_)* | 19.44 ± 4.60 (0) | 20.30 ± 5.59 (0) | 0.25 |
| Time to EEG (h): day two, *M* ± *SD (N_missing_)* | 43.49 ± 4.65 (0) | 44.16 ± 5.87 (0) | 0.24 |
| Temperature (°C): day one, *M* ± *SD (N_missing_)* | 35.75 ± 1.13 (0) | 35.96 ± 0.83 (0) | 0.45 |
| Temperature (°C): day two, *M* ± *SD (N_missing_)* | 37.01 ± 0.95 (0) | 36.91 ± 0.68 (0) | 0.30 |
| FOUR score: day one, *M* ± *SD (N_missing_)* | 4.08 ± 1.78 (7) | 2.79 ± 1.84 (11) | **<0.005** |
| FOUR score: day two, *M* ± *SD (N_missing_)* | 7.06 ± 4.06 (0) | 3.92 ± 2.65 (0) | **<0.0005** |
| EEG reactivity: day one, *N (N_missing_)* | 31 (6) | 5 (10) | **<0.0005** |
| EEG reactivity: day two, *N (N_missing_)* | 35 (7) | 11 (10) | **<0.0005** |
| EEG discontinuity: day one, *N (N_missing_)* | 14 (1) | 33 (6) | **<0.0005** |
| EEG discontinuity: day two, *N (N_missing_)* | 2 (5) | 16 (10) | **<0.0005** |
| Electrographic epileptic activity: day one, *N (N_missing_)* | 0 (1) | 17 (7) | **<0.0005** |
| Electrographic epileptic activity: day two, *N (N_missing_)* | 1 (5) | 11 (10) | **<0.0005** |

*N* refers to the number of patients for which the statistic is computed and *N_missing_* refers to the number of patients without values. ROSC = Return of spontaneous circulation; CA = Cardiac Arrest; M = mean; SD = standard deviation. Note that for the etiology of cardiac arrest, all remaining patients had a pulmonary etiology.

**Supplementary Table 3.** **Demographics and clinical characteristics of FO patients correctly versus incorrectly predicted on day one (i.e. ‘Above’ and ‘Below’ the threshold of the EEG power spectra value for outcome prediction), in the subset of FO patients recorded on both days (N = 45).**

|  | **Power Spectra** | |  |
| --- | --- | --- | --- |
| **FO Day one** | **Above** | **Below** | **p-value** |
| *N* | 18 | 27 |  |
| Time to ROSC (min), *M* ± *SD (N_missing_)* | 23.44 ± 15.90 (0) | 15.19 ± 8.97 (0) | 0.07 |
| Age (y), *M* ± *SD (N_missing_)* | 58.28 ± 14.28 (0) | 67.00 ± 11.86 (0) | 0.07 |
| Gender (male), *N (N_missing_)* | 17 (0) | 22 (0) | 0.38 |
| Cardiac etiology of CA, *N (N_missing_)* | 14 (0) | 20 (0) | 1.00 |
| Pupillary reflexes, *N (N_missing_)* | 15 (3) | 23 (2) | 0.52 |
| Corneal reflexes, *N (N_missing_)* | 14 (3) | 18 (2) | 0.22 |
| Motor response, *N (N_missing_)* | 12 (3) | 15 (2) | 0.30 |
| Time to EEG (h), *M* ± *SD (N_missing_)* | 18.28 ± 5.02 (0) | 20.22 ± 4.22 (0) | 0.27 |
| Temperature (°C), *M* ± *SD (N_missing_)* | 35.72 ± 0.96 (0) | 35.77 ± 1.24 (0) | 0.74 |
| FOUR score, *M* ± *SD* *(N_missing_)* | 3.73 ± 1.79 (7) | 4.30 ± 1.77 (11) | 0.30 |
| EEG reactivity, *N (N_missing_)* | 12 (3) | 19 (3) | 1.00 |
| EEG discontinuity, *N (N_missing_)* | 3 (1) | 12 (0) | **<0.05** |
| Electrographic epileptic activity, *N (N_missing_)* | 0 (1) | 0 (0) | 1.00 |

*N* refers to the number of patients for which the statistic is computed and *N_missing_* refers to the number of patients without values. ROSC = Return of spontaneous circulation; CA = Cardiac Arrest; M = mean; SD = standard deviation. Note that for the etiology of cardiac arrest, all remaining patients had a pulmonary etiology.
